# Effect of arginase II on L-arginine depletion and cell growth in murine cell lines of renal cell carcinoma

**DOI:** 10.1186/1756-8722-1-14

**Published:** 2008-09-25

**Authors:** David J Tate, Derek J Vonderhaar, Yupanqui A Caldas, Toye Metoyer, John R Patterson, Diego H Aviles, Arnold H Zea

**Affiliations:** 1 Stanley S. Scott Cancer Center, LSUHSC, New Orleans, USA; 2 Division of Renal Diseases and Hypertension, UCDHSC, Denver, Colorado, USA; 3 Morehouse School of Medicine, Atlanta GA, USA; 4 Division of Pediatric Nephrology, Children's Hospital, New Orleans, LA, USA; 5 Microbiology Immunology and Parasitology, LSUHSC, New Orleans, LA, USA

## Abstract

**Background:**

L-arginine is the common substrate for the two isoforms of arginase. Arginase I, highly expressed in the liver and arginase II mainly expressed in the kidney. Arginase I-producing myeloid derived suppressor cells have been shown to inhibit T-cell function by the depletion of L-arginine. On the other hand, arginase II has been detected in patients with cancer and is thought to metabolize L-arginine to L-ornithine needed to sustain rapid tumor growth; however its role in L-arginine depletion is unclear. Thus, in tumor biology, L-arginine metabolism may play a dual role in tumor growth and in the induction of T cell dysfunction. Therefore, we studied in murine renal cell carcinoma (RCC) cell lines, the effect of arginase II on tumor cell proliferation and L-arginine depletion. The effect of arginase inhibitors on cell proliferation was also tested.

**Methods:**

Three murine renal cell carcinoma (mRCC) cell lines were tested for the presence of arginase. nor-NOHA, an arginase inhibitor was used to substantiate the effect of arginase on cell growth and L-arginine depletion. Amino acid levels were tested by HPLC.

**Results:**

Our results show that mRCC cell lines express only arginase II and were able to deplete L-arginine from the medium. Cell growth was independent of the amount of arginase activity expressed by the cells. nor-NOHA significantly (*P *= 0.01) reduced arginase II activity and suppressed cell growth in cells exhibiting high arginase activity.

The depletion of L-arginine by mRCC induced the decrease expression of CD3ζ a key element for T-cell function.

**Conclusion:**

The results of this study show for the first time that arginase II produced by RCC cell lines depletes L-arginine resulting in decreased expression of CD3ζ. These results indicate that RCC cell lines expressing arginase II can modulate the L-arginine metabolic pathway to regulate both cell growth and T-cell function. Blocking arginase may lead to a decrease in RCC cell growth and aid in restoring immune function by increasing L-arginine availability for T-cell use. Understanding the interplay between arginase II and its interaction with the immune system may provide future therapeutic benefits to treat patients with RCC.

## Background

L-arginine is a basic amino acid that plays a central role in multiple systems including the immune system [1-3]. Two independent enzymatic pathways, arginase and inducible nitric oxide synthase (iNOS), regulate L-arginine availability. L-arginine is metabolized to L-ornithine and urea by arginase, which is important in the urea cycle and in the biochemical pathways essential for cell proliferation [4,5]. Arginase has two isoforms: arginase I, a cytosolic enzyme found predominantly in hepatocytes, erythrocytes, and granulocytes [6-8] and arginase II, found in the mitochondria of many different tissues, including kidney, brain, and prostate [6,9,10]. Arginase I, is primarily involved in the detoxification of ammonia and urea synthesis, whereas arginase II is involved in the synthesis of L-ornithine, L-proline, and L-glutamate [11].

Several studies have shown that decreased plasma L-arginine levels and nitric oxide (NO) metabolites induced by trauma are associated with an increase in arginase I expression in mononuclear immune cells [12,13], suggesting that L-arginine may have an effect on metabolic processing in the immune system. In patients with renal cell carcinoma (RCC), we have demonstrated that arginase I-producing myeloid suppressor cells depletes plasma L-arginine levels that decreases the expression of T-cell CD3ζ chain [14]. Arginase II on the other hand, is constitutively expressed in normal kidney [15] and its activity shown to be increased in breast, colon, and prostate cancer [16-18]. This activity may sustain the high demand of polyamines necessary for tumor growth. Even though, the depletion of L-arginine has been exclusively attributed to arginase I [19-21], the potential role of arginase II in L-arginine depletion has not been taken into detailed consideration. Likewise, the role of arginase II in tumor growth and in the induction of T-cell dysfunction has not been determined.

In this study we demonstrate for the first time that only arginase II is produced by murine renal cell carcinoma (mRCC) cell lines and that high enzyme levels, specifically depletes extra cellular L-arginine. This amino acid deprivation induces the downregulation of CD3ζ expression in co-cultured Jurkat T-cells. Arginase inhibitors significantly suppressed cell growth in cell lines presenting high arginase II activity.

## Methods

### Tissue culture medium

Complete tissue culture medium consisted of RPMI-1640 containing 1,140 μM L-arginine and supplemented with 10% fetal calf serum (Hyclone, Logan, UT), 25 mM HEPES, 4 mM L-glutamine, and 100 units/mL penicillin/streptomycin, 1 mM non-essential amino acids, and 1 mM sodium pyruvate. All other reagents were purchased from Lonza Walkersville Inc., Walkersville, MD.

### Cell culture

For this study we used mRCC cell lines SIRCC-1.2 (CL-2) and SIRCC 1.19 (CL-19), both of which are sub-clones derived from a streptozotocin-induced kidney tumor [22] and Renca. All of the cell lines were kindly provided by Dr. Robert H. Wiltrout (NCI). Cells were cultured at 37°C in complete media and subcultured every 3 days. Experiments were prepared by plating 300,000 cells in six-well plates and allowed to attach for 24 hours. Media was changed (Time 0) to perform all of the experiments. The cells were harvested at 24, 48, and 72 hours using 0.5% Trypsin/EDTA (Sigma, St. Louis, MO) and lysed with a Triton-based buffer [23] to obtain cytoplasmic extracts to test immediately for arginase activity. Protein concentration was determined by the BCA (bicinchoninic acid) protein assay kit (Pierce Biotechnology Inc., Rockford, IL). Lysates were stored at -70°C until used for Western blots.

### Arginase activity

Freshly prepared cytoplasmic extracts from cultured mRCC cells were tested for arginase activity by the conversion of L-arginine to L-ornithine (nanomoles/10^6^cells/hr), as described elsewhere [24].

### Western blot

Twenty-five micrograms of cytoplasmic extract were electrophoresed in 14% Tris-glycine gels (Invitrogen, Carlsbad, CA) and transferred to polyvinylidiene difluoride (PVDF) membranes (Invitrogen). Immunoblotting were performed with antibodies for arginase I or arginase II (1:200, Santa Cruz Biotech, Santa Cruz, CA). Detection was achieved by horseradish peroxidase-conjugated antibodies (1:3000, Santa Cruz) and an enhanced chemiluminescent kit (ECL, GE Healthcare, Piscataway, NJ). Arginase protein levels were visualized on X-OMAT AR films (Kodak, Rochester, NY).

### Reverse transcriptase polymerase chain reaction (RT-PCR)

Total RNA from 1 × 10^6 ^cells were extracted using TRIzol (Invitrogen), treated with DNase I (Invitrogen), and reverse transcribed using Superscript II (Invitrogen). PCR amplification was done using primers for mouse arginase I, arginase II, and β-actin as follow: Arginase I forward 5'-CAG AAG AAT GGA AGA GTC AG-3', reverse 5'-CAG ATA TGC AGG GAG TCA CC-3', Arginase II forward 5'-TGA TTG GCA AAA GGC AGA GG-3', reverse 5'-CTA GGA GTA GGA AGG TGG TC-3', and β-actin forward 5'-CCA GAG CAA GAG AGG TAT CC-3', reverse 5'-CTG TGG TGG TGA AGC TGT AG-3'. The expected sizes of amplified fragments were arginase I, 250 bp; arginase II, 310 bp; and β-actin, 436 bp. PCR products were visualized in ethidium bromide agarose gels.

### Amino acid detection

High performance liquid chromatography (HPLC) was conducted on deproteinized supernatants labeled with *O*-phtaldialdehyde (OPA). Analytes were eluted with 100 mM sodium acetate buffer, pH 5.0, with a linear gradient consisting of methanol (80%) and acetonitrile (80%). The analytes in the sample were calculated on the basis of standard curves of known amounts.

### Proliferation assays

Cells (1 × 10^4^/well/1 mL) were plated in 24-well plates and allowed to adhere for 24 hours. The cells were treated with different concentrations of N^ω^-Hydroxy-nor-L-arginine (nor-NOHA), 0.5 mM, 1 mM, and 2 mM to determine the optimal conditions to suppress cell growth, or cultured without the inhibitor to be used as controls. The cultures were pulsed once with [^3^H]-thymidine (1.0 μCi, Perkin Elmer Life Sciences, Boston, MA) and tested for [^3^H] incorporation at 24, 48, and 72 hours using a TOPCOUNT Microplate Scintillation Counter (Packard, Meridien, CT). Cell viability was checked by trypan blue exclusion at each time point. Each condition was tested in triplicate.

### Expression of CD3ζ by co-cultured Jurkat T-cells

To determine the effect of L-arginine deprivation on the expression of CD3ζ, mRCC cell lines (600,000 cells/well) were cultured in six-well plates with 5 mL of complete media for 24 hours. 5 × 10^5 ^Jurkat T-cells (ATCC, Manassas, VA) were then added to the upper chamber of a trans-well system (Falcon-BD, San Jose, CA) and co-cultured for 24, 48, and 72 hours. CD3ζ expression was determined by flow cytometry as described elsewhere [25]. Jurkat cells cultured in complete media were used as controls. L-arginine levels were determined in the supernatants by HPLC.

### Statistical analysis

Statistical analysis was calculated by Student's *t*-test using the Graph Pad Prism 3.0 statistical program (GraphPad Software Inc., San Diego, CA). *P *< 0.05 was taken to indicate statistical significance.

## Results

### Arginase expression in mRCC cell lines

**Figure 1:**
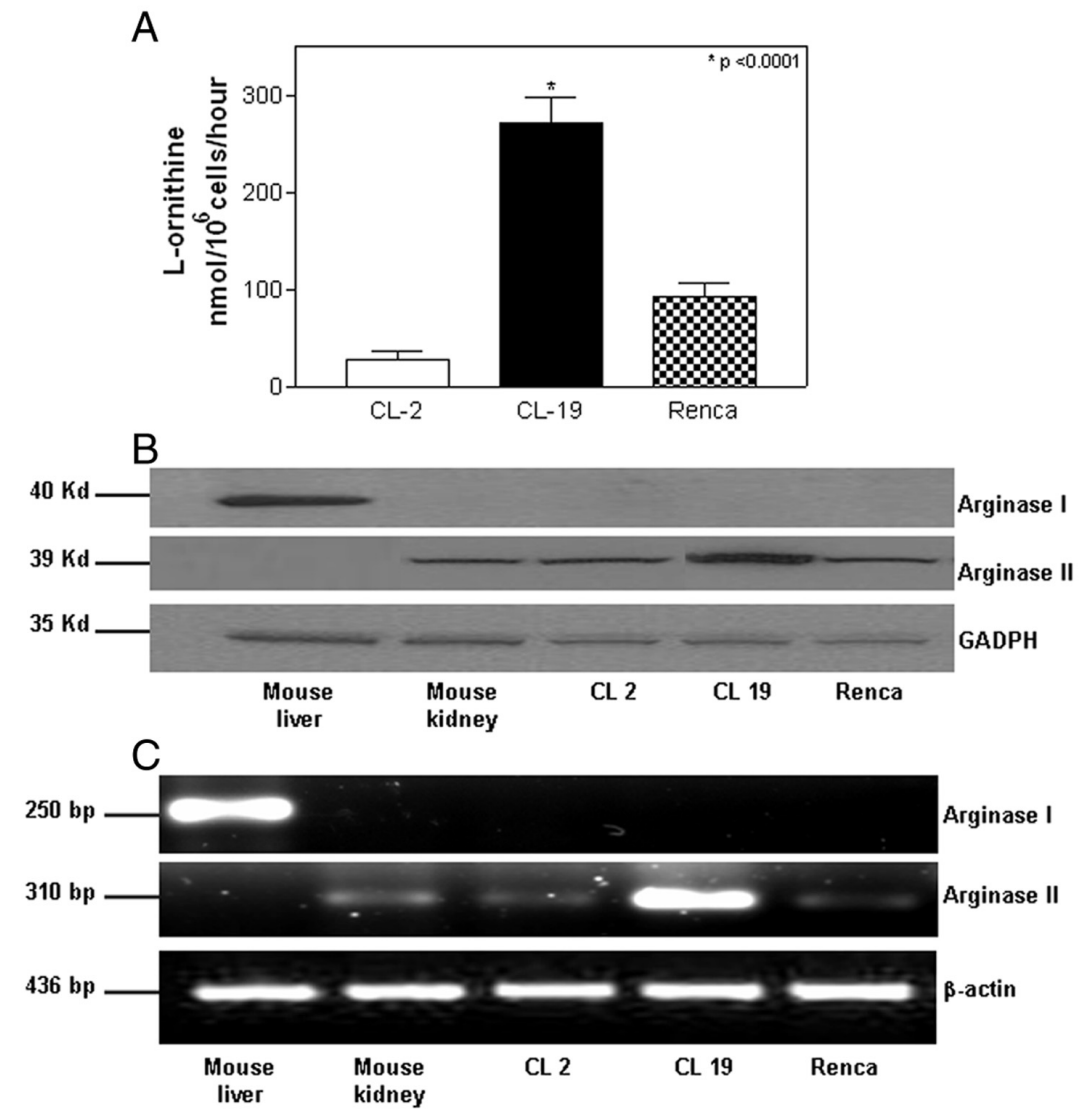
**Arginase II expression in mRCC cell lines**. (A) After 48 hours in culture, CL-19 cells presented significantly more arginase activity (*P < 0.0001) than did either CL-2 or Renca cells. Similar results were found after 72 hours in culture. (B) Twenty five micrograms of protein were tested for arginase I and arginase II expression by Western blot analysis. Normal mouse liver and kidney were used as positive controls for arginase I and arginase II respectively, whereas GAPDH was used as house keeping protein. (C) Total RNA from CL-2, CL-19 and Renca cells were obtained by TRIzol extraction and 1 μg of RNA was tested for arginase I, arginase II, and β-actin by RT-PCR. DNA fragment sizes generated by RT-PCR: arginase I, 250 bp; arginase II, 310 bp; and β-actin, 436 bp. These data are from a single experiment that is representative of five separate experiments.

First, we investigated the enzymatic activity of arginase in the mRCC cell lines CL-2, CL-19, and Renca. CL-19 cell line had the highest arginase activity, which was 3.0-fold greater than the Renca and 9.8-fold greater than the CL-2 cell line (Figure 1A). Using specific antibodies for arginase I and arginase II, we found that the cell lines only expressed detectable levels of arginase II, and not arginase I (Figure 1B). By RT-PCR, we observed arginase II gene expression in all 3 of the mRCC cell lines, but not arginase I gene expression (Figure 1C). Arginase II mRNA expression was greatest in CL-19 compared to Renca and CL-2 cell lines. The data show a direct association amongst arginase II mRNA expression, protein expression, and enzymatic activity.

### Arginase II produced by the CL-19 cell line depletes extra cellular L-arginine

**Figure 2:**
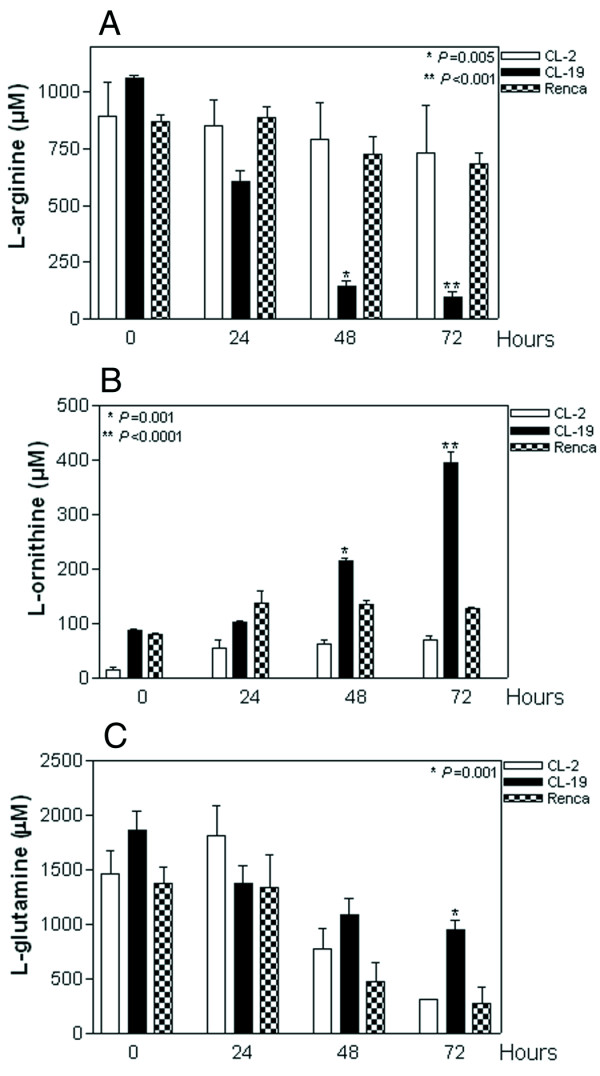
**L-arginine, L-ornithine and L-glutamine levels**. Tissue culture supernatants from CL-2, CL-19, and Renca cells were collected at 24, 48, and 72 hours. They were analyzed by HPLC after deproteinization with methanol and derivatization with OPA for (A) L-arginine and (B) L-ornithine and (C) L-glutamine. Standards of L-arginine, L-ornithine and L-glutamine in methanol were run with each experiment. Results are expressed as means ± SE of duplicate determinations from four independent experiments. (* *P *= 0.005. ** P < 0.0001 significant differences for CL-19 compared to the other cell lines).

The effect of arginase II on L-arginine, L-ornithine, and L-glutamine content in the conditioned culture medium was assessed by HPLC. The CL-19 cell line, which expressed high levels of arginase II, depleted the media L-arginine concentration by about 50% at 24 hours (*P *= 0.005, Figure 2A) and about 90% at 48 and 72 hours (*P *< 0.001) when compared to media controls. L-arginine levels remained unchanged in CL-2 and Renca cultures throughout the experimental time points. Concomitantly, there was a significant increase in L-ornithine production by CL-19 after 48 and 72 hours (*P *= 0.001 and *P *< 0.0001) compared to CL-2 and Renca cell lines in which the levels did not change significantly at any time point (Figure 2B). All cell lines also depleted the culture supernatants of L-glutamine at the same rate during the first 24 hours. However, at 72 hours, the depletion of L-glutamine was significantly higher in CL-2 and Renca (*P *= 0.001 and *P *= 0.016) than in CL-19 (Figure 2C).

### Role of arginase II and effect of nor-NOHA on mRCC cell proliferation

We assessed whether arginase II could play a role in the proliferation of the three different mRCC cell lines. The cell lines were cultured for 24 hours and the media replaced with 0.5, 1 and 2 mM of nor-NOHA, pulsed once with [^3^H]-thymidine and tested for [^3^H] incorporation after 24, 48, and 72 hours in culture. At 72 hours in culture a concentration of 2 mM nor-NOHA was able to significantly suppress cell growth in the high arginase producer CL-19 cell line. No significant effect on cell growth suppression was observed with the lower concentrations of nor-NOHA (data not shown). Therefore, we used 2 mM nor-NOHA for the rest of the experiments.

**Figure 3:**
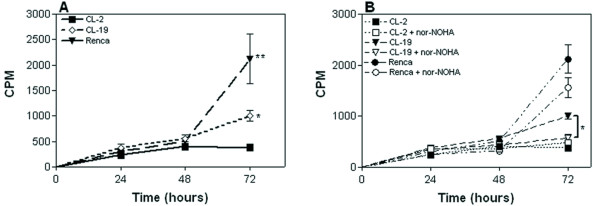
**Effect of arginase inhibitor nor-NOHA on cell proliferation**. (A) Proliferation of CL-2, CL-19 and Renca cells was assessed by [^3^H]-thymidine incorporation at 24, 48, and 72 hours in culture. At 72 hours, the growth rates for CL-19 and Renca cells were significantly greater than CL-2 (* *P *= 0.009 and ** *P *= 0.003 respectively). (B) nor-NOHA (2 mM) and [^3^H]-thymidine were added at the same time and cell proliferation was determined at 24, 48, and 72 hrs. Cultured cells without the inhibitor were used as controls. Only CL-19 proliferation was significantly inhibited (* *P *= 0.010) compared to the untreated control cells. Results are expressed as CPM means ± SE of triplicate determinations from five independent experiments.

When the 3 cell lines were cultured in presence of 2 mM nor-NOHA, they incorporated [^3^H]-thymidine at the same rate during the first 48 hours. However at 72 hours, significant differences in cell proliferation among the lines were apparent. CL-2 proliferation was significantly lower (*P *= 0.003) compared to CL-19, which presented the highest arginase activity. Interestingly, the Renca cell line, which had intermediate arginase activity, had the highest proliferation rate compared to the CL-19 (*P *= 0.01) and CL-2 (*P *= 0.003) cell lines (Figure 3A). The higher L-glutamine consumption observed in Renca cells (Figure 3C) suggested that this line may utilize a different pathway for its cellular growth, bypassing arginase for the production of L-ornithine.

We then tested the effect of the arginase inhibition by nor-NOHA (2 mM) on cell proliferation at 24, 48, and 72 hours. Growth of CL-19, which had the highest level of arginase II activity, was significantly inhibited (*P *= 0.017, Figure 3B). In contrast, nor-NOHA had no significant effects on the growth rates of the low arginase producer cell lines CL-2 (*P *= 0.14) and Renca (*P *= 0.07). Cell viability of the cells was > 95% at the different points and conditions of the experiments.

### nor-NOHA blocks arginase activity and L-arginine consumption in CL-19 cell line

**Figure 4:**
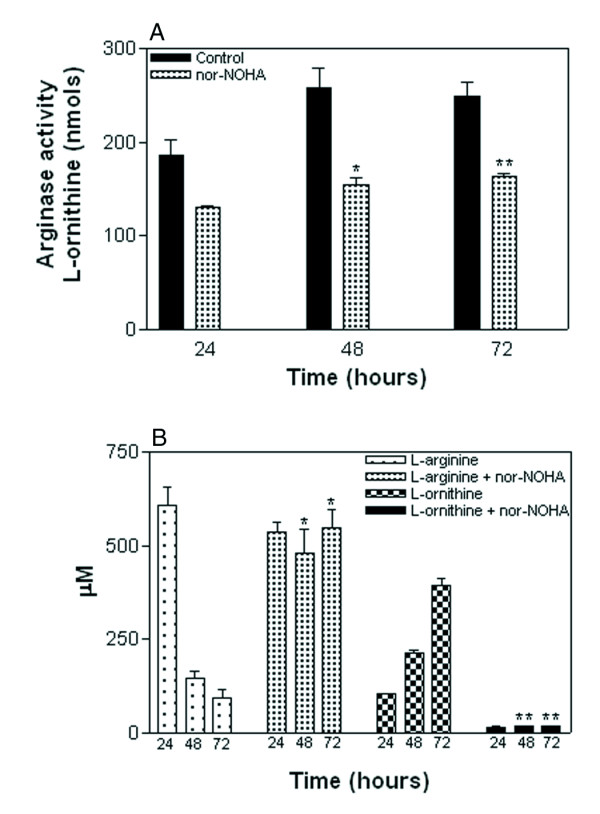
**Effect of nor-NOHA on arginase activity and amino acid levels**. (A) Significant arginase inhibition was observed in cell lysates of CL-19 cultures treated with nor-NOHA (2 mM) after 48 (*P = 0.002) and 72 hours (** *P *= 0.001) as compared to untreated cells. (B) Effect of nor-NOHA in inhibiting both L-arginine (μM) depletion (*P = 0.001) and L-ornithine (μM) production (**P < 0.0001) in the supernatants of CL-19 cultures, as compared to CL-19 untreated cultures. Results are expressed as means ± SE of duplicate determinations from four independent experiments.

Since nor-NOHA significantly inhibited cell proliferation of CL-19, we wanted to test the effect of this inhibitor on arginase activity and L-ornithine production. Arginase activity increased in this cell line over the time of the experiments. When 2 mM of nor-NOHA was added to the cultures, significant reduction in arginase activity occurred at 48 and 72 hours (*P *= 0.002 and *P *= 0.001 respectively) (Figure 4A). Importantly, independent of the amount of arginase produced by this cell line, the effect of arginase inhibition by nor-NOHA was similar at all time points tested. The inhibition of arginase activity in CL-19 by nor-NOHA significantly blocked (*P *= 0.0001) the depletion of L-arginine as well as the accumulation of L-ornithine (*P *< 0.0001) after 48 hours compared to CL-19 cultures without the inhibitor (Figure 4B). We did not observe significant changes in arginase inhibition, L-arginine depletion and L-ornithine production when nor-NOHA was added to cultures with CL-2 and Renca cells (data not shown).

### L-arginine depletion by arginase II induces CD3ζ downregulation in Jurkat T-cells

**Figure 5:**
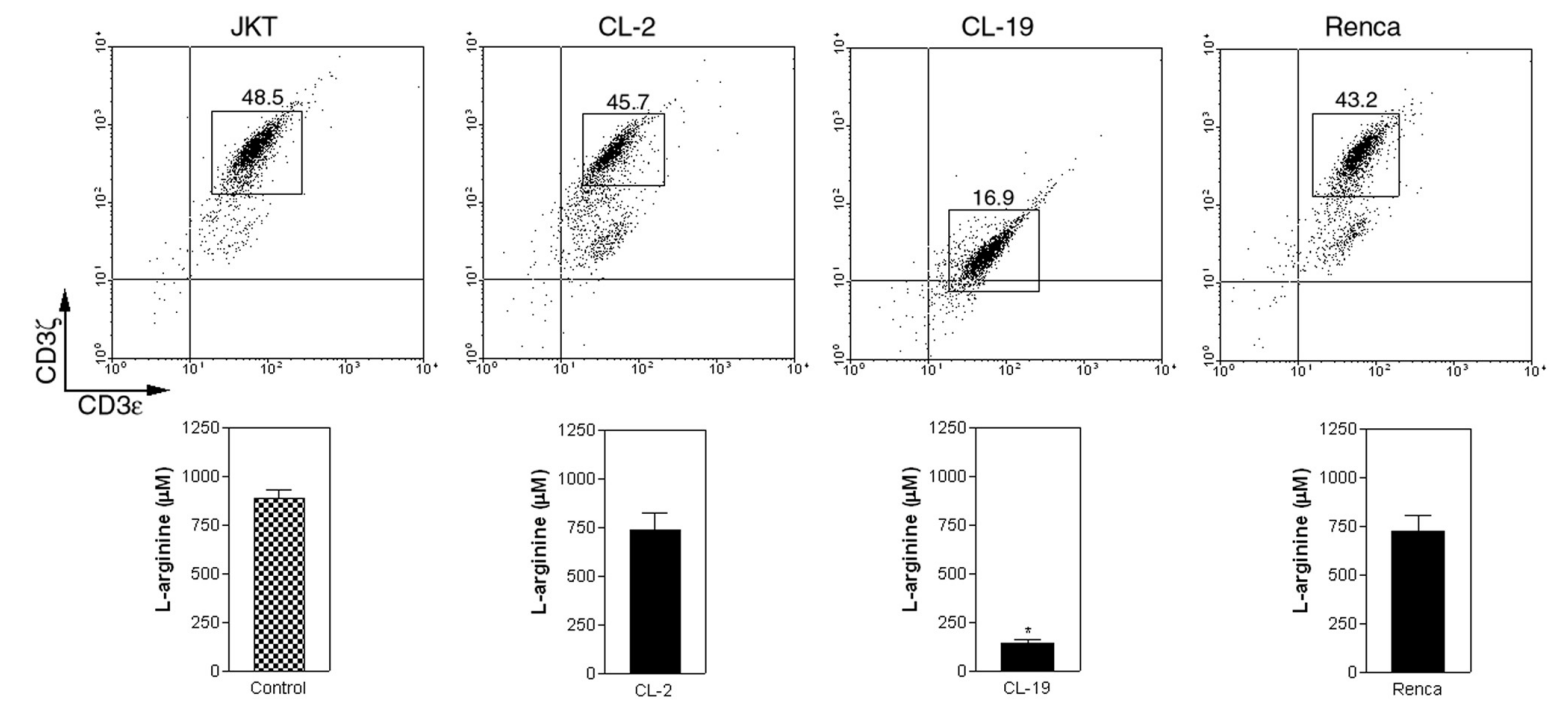
**L-arginine deprivation and its effect on CD3ζ expression**. (Upper panel) Expression of CD3ζ in Jurkat T-cells (JKT) cultured for 48 hours alone (control) or co-cultured in trans-wells with CL-2, CL-19, or Renca cell. Differences in CD3ζ expression were measured by mean fluorescence intensity. (Lower panel) L-arginine levels in supernatants after 48 hours of co-culture of Jurkat cells with the cell lines. Levels of L-arginine were significantly lower (* *P *< 0.001) in CL-19 co-cultures than in Jurkat control or in co-cultures with CL-2 and Renca cell lines. Data shown is representative of a single experiment at 48 hours in culture.

Jurkat T-cells rapidly lose CD3ζ in absence of L-arginine. Therefore, we tested if L-arginine depletion by mRCC arginase II had any effect on CD3ζ expression in trans-wells co-cultured Jurkat T-cells. At 24 hours in co-culture with any of the mRCC cell lines, Jurkat T-cells did not show any significant reduction on CD3ζ expression. However, after 48 hours Jurkat T-cells co-cultured with CL-19 had a dramatic decrease in the expression of CD3ζ as compared to Jurkat controls (MFI: 16.9 and 48.5 respectively, Figure 5). The decreased expression of CD3ζ in Jurkat T-cells paralleled the significant depletion of L-arginine (*P *= 0.03) in the co-cultured CL-19 compared to the Jurkat control (Figure 5 lower panel). In contrast, the expression of CD3ζ in Jurkat T-cells co-cultured with CL-2 or Renca was similar to that expressed in the Jurkat control, where the levels of L-arginine remained unchanged (Figure 5).

## Discussion

Our major objective was to assess whether arginase II was able to deplete L-arginine from the tissue culture supernatants of murine renal cell carcinoma cell lines and determine their effect on cell proliferation. In adult mammals, the majority of endogenous L-arginine is synthesized from citrulline in the kidney and released to systemic circulation where it is catabolized by arginase I or arginase II [4,11]. Therefore, the study of the L-arginine metabolic pathway in mRCC cell lines provide us with a good model to better understand the biology of renal carcinoma.

Western blot and RT-PCR analyses confirmed that arginase activity from mRCC cell lines was attributable solely to arginase II and not to arginase I. This is an important finding, since most studies have demonstrated that only arginase I produced by tumor cells, macrophages, smooth muscle and endothelial cells [26-29] is capable of depleting L-arginine which results in the induction of T-cell dysfunction [19,30]. The role of arginase II on L-arginine metabolism in disease and cancer has been quite underestimated, especially taking into account its wide tissue distribution and its role in polyamine production. Previous studies have shown that the expression of either arginase I or arginase II plays a key role in polyamine synthesis and cell proliferation [31]. Although the three cell lines used in this study were all derived from kidney tumors, they had very different arginase II activities. CL-2 and CL-19, both derived from renal tumors induced by streptozotocin, had low and high arginase II activities, respectively. The Renca cell line, derived from a spontaneous renal tumor had intermediate activity. These three lines provide us with an ideal model to study the biology of RCC with regard to L-arginine consumption, L-ornithine production, and cell proliferation. We demonstrate for the first time that arginase II produced by the high arginase RCC cell line CL-19 dramatically depletes L-arginine from the tissue culture supernatants at 48 hours with a concomitant increase in L-ornithine production.

In contrast, the cell lines CL-2 and Renca, both of which expressed low levels of arginase II compared to CL-19 did not deplete L-arginine significantly; nor did they increase the levels of L-ornithine sufficiently to promote growth. Instead, we observed that CL-2 and Renca cells utilize L-glutamine at higher rates than CL-19, suggesting that this could be a possible mechanism used by these cells to convert L-glutamine to glutamate, bypassing arginase for the production of L-ornithine as described previously in murine macrophages and human monocytes [32]. It is likely that CL-2 and Renca cells do not need arginase to make L-ornithine because they utilize L-glutamine to produce the necessary amount of L-ornithine needed for their cell growth. This observation indicates that arginase II is important for CL-19 growth but not for CL-2 and Renca cells due to the positive effect of nor-NOHA in suppressing cell growth in CL-19. We used nor-NOHA in our experiments because it has been demonstrated that nor-NOHA is a potent and selective inhibitor of arginase [33] in contrast to NOHA which is a key intermediate product in the biosynthesis of nitric oxide by L-arginine. We were expecting to have a greater arginase inhibition by nor-NOHA in our cultures similar to those observed when NOHA was used to inhibit cell proliferation in cell lines from breast, colon, prostate and endothelial cells as previously reported [16,17,34,35]. This may be due to the fact that these cell lines can use L-arginine to synthesize NOHA from arginase, then increasing its inhibitory effect. In contrast, it is also possible the growth of renal cell carcinoma cells is arginase II independent resulting in the low inhibitory effect of nor-NOHA.

At 48 hours in culture, CL-19 cells significantly depleted L-arginine from the culture supernatant; however, these cells continued growing at the same rate up to 120 hours in the absence of the amino acid (data not shown). L-arginine deprivation should promote the death of CL-19 cells, as reported previously to occur in other cancer cells lines [36,37], indicating that these cells are more adept at circumventing L-arginine deficiency by increasing the recycling efficiency from L-ornithine to citrulline to convert L-arginine fast enough to sustain relatively normal tumor cell growth rate as previously shown [38]. Since RCC cells have a strong dependence for L-arginine [39], our laboratory is currently studying whether or not these cells are utilizing L-glutamine or citrulline as the source for L-arginine synthesis.

L-arginine is a non-essential amino acid that plays a central role in several biological systems including the immune response. Paradoxically, L-arginine deprivation can cause tumor cell death as well as T-cell dysfunction. The loss of CD3ζ is the only arginase-triggered mechanism described so far that has proven to have direct relevance to T-cell function [40,41]. It has been previously shown that Jurkat T-cells cultured in medium lacking L-arginine showed decreased expression of CD3ζ and decreased cell proliferation [42]. Similar results were obtained when stimulated normal human T-cell lymphocytes were cultured in the absence of L-arginine [25]. In the current experiments, we found that after 48 hours in culture, depletion of L-arginine by CL-19 arginase II activity caused the decreased expression of CD3ζ in co-cultured Jurkat T-cells. Therefore, L-arginine availability can regulate the expression of CD3ζ, an essential component in T-lymphocyte signal transduction and function. L-arginine levels in the serum of normal individuals ranges from 115 μM to 210 μM [4]. Our data show that at 48 hours, the levels of L-arginine in the trans-well tissue culture supernatant was 100 μM, a concentration sufficient to induce a decrease in CD3ζ expression.

Most tumor cells have a great demand for amino acids to support rapid proliferation and L-arginine is the first amino acid depleted faster than other nutrients by normal cell metabolism. Therefore, L-arginine could be a reasonable target of deprivation strategy for the type of tumors with a low recycling efficiency. Taken collectively, these findings, demonstrate that the availability of L-arginine and L-ornithine could be the limiting factor to control cell proliferation. We believe that treating RCC cells downstream from the L-arginine metabolic pathway by blocking polyamine production will have a major impact in suppressing tumor growth. This is supported by the use of DL-α-difluoromethylornithine, which completely blocks the proliferation of these cell lines independent of the presence of L-arginine, L-ornithine and arginase in addition to the promising anti-tumor effect in human tumors [43-45].

Renal cell carcinoma is a malignancy with poor prognosis due to its strong resistance to conventional cancer treatments and frequent metastases. With the standard immunotherapeutic treatment of IL-2 and IFNα for RCC, only 10–20% of the patients respond [46]. This lack of response may be caused by the markedly impaired T-cell function associated with a decreased expression of the CD3ζ receptor. Therefore, it is still desirable to find better approaches to treat RCC. Modulating the L-arginine metabolic pathway by breaking down this amino acid required for tumor cell growth could be a novel approach to control it. The study of the mechanisms by which arginase II activity and L-arginine depletion affect tumor growth will help better understand the biology of RCC and its interaction with the immune system. The results of these studies may provide future therapeutic benefits.

## Conclusion

Arginase II produced by renal cell carcinoma cells can modulate L-arginine levels to regulate both cell growth and T cell function. Blocking arginase may lead to a decrease in RCC cell growth and aid in restoring immune function by blocking the formation of polyamines, thus providing a novel therapeutic advance.

## Competing interests

The authors declare that they have no competing interests.

## Authors' contributions

DJT participated in the design of the study, developed HPLC for the detection of amino acid levels, performed RT-PCR, analyzed the collected data and wrote the manuscript; DJV participated in the study design and conducted western blots and arginase activity assays; YAC participate in the analysis of HPLC data and design and conducted functional assays; TM participated in tissue culture and preparation of cell lysates and RNA extractions; JRP participated in performing arginase activity assays, western blots, amino acid assays and data collection; DHA was involved in the analysis and interpretation of data and critically revised the manuscript; AHZ designed the study, performed flow cytometry assays, analyzed and interpreted the data and wrote the manuscript. All authors read and approved the manuscript.
